# Evaluating the implementation of a multidisciplinary lifestyle intervention for people with severe mental illness in sheltered housing: effectiveness-implementation hybrid randomised controlled trial

**DOI:** 10.1192/bjo.2022.600

**Published:** 2022-11-22

**Authors:** Marij M. C. Smit, Elze de Waal, Diederik E. Tenback, Jeroen Deenik

**Affiliations:** GGz Centraal, Amersfoort, The Netherlands; and Faculty of Social and Behavioural Sciences, Utrecht University, Utrecht, The Netherlands; GGz Centraal, Amersfoort, The Netherlands; and Faculty of Social and Behavioural Sciences, University of Amsterdam, Amsterdam, The Netherlands; Centre for Transcultural Psychiatry (CTP)Veldzicht, Balkbrug, The Netherlands; GGz Centraal, Amersfoort, The Netherlands; and School for Mental Health and Neuroscience, Maastricht University, Maastricht,The Netherlands

**Keywords:** Schizophrenia, sedentary behaviour, exercise, diet, implementation science

## Abstract

**Background:**

Lifestyle interventions can improve health-related outcomes for people with severe mental illness (SMI), but few studies evaluate this potential in everyday settings. After a successful approach in routine inpatient mental healthcare (MULTI), we sought to replicate this multidisciplinary lifestyle-enhancing support in people with SMI living in sheltered housing (MULTI_sh).

**Aims:**

To evaluate the effectiveness and implementation of MULTI_sh (trial registration: NCT03157557).

**Method:**

In an effectiveness-implementation hybrid cluster-randomised controlled trial, six municipalities with sheltered housing facilities in The Netherlands were randomly assigned to MULTI_sh (*n* = 3) or treatment as usual (TAU, *n* = 3). After 12 months, we evaluated effects on metabolic health, sedentary behaviour/physical activity (ActiGraph GT3X+), quality of life (EuroQol 5D, WHOQoL-Bref) and psychopathology (Brief Psychiatric Rating Scale Expanded Version) using multiple regression, adjusting for baseline values and municipalities (intention to treat and per protocol). In addition, implementation fidelity and barriers/facilitators were evaluated (Measurement Instrument for Determinants of Innovation).

**Results:**

Of 177 eligible patients, 74 (42%) could be included in the analyses. Health outcomes did not substantially improve with MULTI_sh (*n* = 45) compared with TAU (*n* = 29). MULTI_sh was not implemented as intended. Most patients and all healthcare professionals believed that patients’ lifestyle should be part of treatment, but implementation was primarily (in)directly hindered by organisational factors (e.g. staff shortages, complexity of participation, lack of time and difficulty getting patients involved).

**Conclusions:**

MULTI_sh was not implemented as intended and no clinical health improvements were found. Organisations are decisive in the success or failure of the implementation of lifestyle interventions for people with SMI. More intensive implementation strategies on this level are warranted in sheltered housing.

Over recent decades, the up to 20 years shortened life expectancy of people with severe mental illness (SMI, e.g. schizophrenia-spectrum, major depressive or bipolar disorder) compared with the general population has remained similar and is mainly caused by comorbid physical conditions, including cardiometabolic diseases.^1–3^ These conditions have a combination of interacting risk factors, including immunometabolic dysregulation,^4,5^ genetic vulnerability,^6^ side-effects of antipsychotic medication,^7^ lower use of somatic care^8^ and an unhealthy lifestyle (i.e. sedentary behaviour, low levels of physical activity, smoking and dietary risks such as malnutrition and low intake of fruit and vegetables).^1^

A growing number of studies focusing on lifestyle interventions in people with SMI show positive improvements in physical and mental health, for example by increasing physical activity and/or improving poor dietary habits.^9–13^ However, there is limited evidence regarding the maintenance and long-term health benefits of lifestyle interventions, especially in naturalistic settings.^12,14,15^ Such evaluations (i.e. effectiveness studies) give a more representative view on effect sizes in everyday healthcare, which may be reduced compared with the ideal conditions in the research context of efficacy studies. Parallel to this, more implementation studies are needed to gain insight into factors influencing the intended implementation of interventions.^16,17^ This informs interpretations of the effects found and suggests strategies to target barriers, thus improving the implementation of the intervention.^18^ The concurrent running of effectiveness and implementation studies speeds up the translation of research into effects on everyday healthcare.^16^

Recently, a multidisciplinary lifestyle-enhancing treatment for in-patients with SMI called MULTI was evaluated 18 months after implementation. MULTI was developed after finding that an integrated approach seemed needed to translate positive attitudes and self-efficacy into behavioural change in the context of the theory of planned behaviour.^19^ MULTI focused on overall physical activation and sustainable behavioural change using daily structure, mainly targeting sedentary behaviour and healthy nutrition and eating habits. The study found improvements in physical activity, metabolic risk factors (e.g. weight, abdominal girth, high-density lipoprotein (HDL) cholesterol), medication use and psychosocial functioning compared with treatment as usual (TAU).^20–22^ Both patients and healthcare professionals were optimistic about the intervention and their role in it.^23^ This is in line with recent studies showing that a multicomponent approach (e.g. physical activity, attention to dietary risks, psychoeducation), multidisciplinary collaboration and engaging healthcare professionals are essential in encouraging a healthy lifestyle in SMI.^1,24,25^

In the context of implementation science, studying the application of a successful intervention in another setting is considered essential when exploring its potential for dissemination and broader adoption.^26^ Sheltered housing facilities for people with SMI are often closely connected to in-patient facilities (e.g. before or after hospital admission) and thus are a logical first setting to extend MULTI to. People with SMI in sheltered housing live more independently and face barriers to adhering to a healthy lifestyle, such as the symptoms of SMI and medication-related side-effects (i.e. metabolic syndrome, sedation and movement disorder).^27^ Also, fear of discrimination and reliance on healthcare professionals to plan and initiate rehabilitation programme activities play a role.^27^

Therefore, we aimed to investigate whether a lifestyle programme based on MULTI (multidisciplinary lifestyle-enhancing support for patients living in sheltered housing, MULTI_sh) could help to improve the lifestyle and, thereby, the health status of people with SMI living in such facilities. In an effectiveness-implementation hybrid randomised controlled trial,^16^ we aimed to evaluate:

- the effectiveness of MULTI_sh on both physical and mental health
- whether MULTI_sh was implemented as intended and to identify associated implementation barriers and facilitators to expedite the translation of research findings into routine care.

## Method

### Design

We used an open-label effectiveness-implementation type-1 hybrid cluster-randomised controlled trial design, testing the effectiveness of MULTI_sh on health-related outcomes while observing and gathering information on implementation.^16^ The study was conducted in the sheltered housing facilities of GGz Centraal (The Netherlands). After baseline measurements (July–October 2017), MULTI_sh was implemented in three sheltered housing locations in April 2018. Follow-up measurements took place 12 months after implementation (March–May 2019). Data on patients were collected from routine screening data (demographic/metabolic health parameters), actigraphy measurement (sedentary behaviour/physical activity) and questionnaires that were completed through semi-structured interviews by trained research assistants (psychopathology, quality of life (QoL), programme implementation fidelity and barriers/facilitators). The 12-month evaluation and extra metabolic health parameters (weight, blood pressure and HbA_1c_), psychopathology and implementation factors were part of an expansion of the originally initiated 6-month evaluation after it was found to be feasible and contributed to the study objectives. Data from healthcare professionals on the implementation of MULTI_sh were collected via an online version of the questionnaire using a unique email link. In addition, three reminder emails were sent at 2-week intervals to all non-responders. The authors assert that all procedures contributing to this work comply with the ethical standards of the relevant national and institutional committees on human experimentation and with the Helsinki Declaration of 1975, as revised in 2008. All procedures involving patients were approved by the Medical Ethical Committee of the Isala Academy (case 170403) and registered at Clinicaltrials.gov (NCT03157557). All participants gave written informed consent.

### Study population

The cohort consisted of 177 individuals (≥18 years) with SMI (schizophrenia spectrum, major depressive or bipolar disorder) living in sheltered housing facilities of GGz Centraal within the Veluwe and Veluwe Valley region of The Netherlands. There were no exclusion criteria for receiving MULTI_sh as it was developed for people with SMI in sheltered housing in which activities were tailored to individual needs and interests (see the paragraph below describing the intervention). For measurements, individuals were included if they lived in one of the six sheltered housing facilities and gave informed consent. They were excluded if they refused measurements or if there was any risk that the measurements could cause (further) worsening of psychiatric symptoms, as evaluated by their respective care team and physician.

### Interventions

The purpose of MULTI_sh was holistic lifestyle change with a focus on decreasing sedentary behaviour, increasing physical activity and improving dietary habits. The intervention consisted of improving daily structure by getting up on time, having joint meals and having an active daily programme consisting of work-related activities (e.g. supporting functions in garden maintenance), sports-related activities (e.g. walking), psychoeducation (e.g. regarding the side-effects of medication) and training in daily living skills (e.g. cooking). Additionally, if necessary, existing policies were reviewed and adjusted (e.g. about eating together and getting groceries by foot/cycle or car). Given the heterogeneity in illness severity, capabilities and interests in the individuals and in the sheltered housing facilities as a whole, the content and intensity of the day-to-day programme were tailored to the particular sheltered housing facility and individual patients with the aim of achieving sustainable change. In line with MULTI in in-patient facilities,^23^ MULTI_sh was based on a ‘change from within’ principle, meaning that the aim was to change patients’ programmes and the team culture within the staffing and resources available in routine care. At each intervention site, a mental healthcare professional with secondary or higher vocational education in mental healthcare (e.g. nurse or social worker) was appointed to coordinate the improvement of daily structures and activity programme in cooperation with the team and by connecting with qualified experts (e.g. exercise professionals and dietitians in the community) to support and supervise this.

Participants who received TAU continued their regular treatment, mainly consisting of a less structured day programme and medication, excluding any extra support in lifestyle interventions or adjustments.

### Outcomes

#### Metabolic health

Metabolic health was the primary outcome measure, represented by abdominal girth measured to the nearest 0.1 cm, on bare skin, across the umbilicus, halfway between the iliac crest and lowest rib in a standing position. Additionally, data on weight (without shoes in an upright position to the nearest 0.1 kg), blood pressure, triglycerides, fasting glucose, HbA_1c_ and total and HDL cholesterol were part of routine somatic screening.

#### Sedentary behaviour and physical activity

Sedentary behaviour and physical activity were measured using the triaxial ActiGraph GT3X+ accelerometer. Detailed procedures and settings used for the baseline and follow-up measurements are described elsewhere.^20,28^ The ActiGraph was worn on the right hip for five consecutive days (Wednesday 09.00 h until Sunday 23.59 h) except for activities involving water and sleeping. Each data-set had the same time frame of 09.00–22.00 h to make the data comparable between participants. To avoid significant drop-out of data, a wear time of a minimum of 6 h a day for at least 3 days was the criterion for valid measurement.^28^ Data were analysed using the ActiLife 6.8.0 software (www.actigraph.nl) and converted into average total activity counts per hour (TAC/h). TAC/h was standardised to facilitate interpretation. The GT3X+ has a high inter- and intra-instrumental reliability and validity.^29,30^

#### Psychopathology

The Dutch version of the Brief Psychiatric Rating Scale Expanded Version (BPRS-E)^31^ was used to evaluate general psychopathology. The BPRS-E consists of 24 items measuring the severity of psychopathology, including schizophrenia and related psychotic disorders. Items are scored from 1 (not present) to 7 (very serious) and divided into four subdomains: manic arousal/disorganisation, depression/anxiety, positive symptoms and negative symptoms. The BPRS has been found to be sufficiently valid and reliable.^32^

#### Quality of life

Quality of life (QoL) was assessed using the Dutch versions of the EuroQol 5D (EQ-5D)^33^ and domains of the World Health Organization Quality of Life Brief Version (WHOQOL-Bref).^34^ The EQ-5D is a simple generic questionnaire measuring five dimensions of health (mobility, self-care, usual activities, pain/discomfort and anxiety/depression) rated from 1 (no problems) to 3 (extreme problems) and has a reasonable validity, reliability and feasibility in people with schizophrenia.^35,36^ The WHOQOL-Bref contains 24 items distinguishing physical, psychological, social and environmental QoL. Items were scored from 1 (not at all/very dissatisfied/very poor/never) to 5 (completely/very satisfied/very good/always) and transformed into domain scores ranging from 4 to 20 according to WHO guidelines.^37^ The WHOQOL-Bref has good reliability, content and construct validity, and sensitivity for assessing QoL in psychiatric patients in general^38^ and specifically patients with schizophrenia.^39,40^

#### Implementation

Implementation fidelity (i.e. the extent to which the intervention was implemented as intended, measured according to a predefined protocol)^41^ and barriers and facilitators were measured using the Measurement Instrument for Determinants of Innovation (MIDI).^42^ The MIDI comprises four subscales, measuring 29 determinants for implementation related to the intervention itself (*n* = 7), the users (healthcare professionals/patients, *n* = 11), the organisation (*n* = 10) and sociopolitical context (*n* = 1) on a five-point scale (totally disagree–totally agree). The questionnaire was used in the previous MULTI study in in-patient facilities and a report for that study gives detailed procedures.^23^ According to the MIDI instruction guide, questions were tailored to the context of sheltered housing facilities (e.g. available disciplines in the context of social support). For patients, we limited the number of items on this questionnaire to those relevant from their perspective, to increase the feasibility and prevent unnecessary burden. As a pragmatic proxy of implementation fidelity, we used the descriptive norm determinant (i.e. how well colleagues implemented MULTI_sh), which was asked in relation to the predefined description of MULTI_sh.

### Sample size

To calculate the sample size, we used the effect size of abdominal girth decrease in the previous intervention study (Cohen's d = 0.51 cm)^20^ in a similar multiple regression analysis corrected for baseline values on age, diagnosis and illness severity. To detect the same effect in the current study with a minimum 80% power as a benchmark for a fair test, a 5% significance level and an expected response rate of 73% based on previous baseline measurements in in-patients with SMI,^28^ a sample size of 168 patients was required (two groups of 84).

### Randomisation and masking

Cluster randomisation was carried out at the level of municipalities (*n* = 6) by an independent research assistant. To ensure equal numbers in both conditions, municipalities were paired based on their number of inhabitants in the sheltered housing facilities. One municipality out of each pair was randomly assigned to receive the intervention. The other municipality out of each pair continued to receive TAU. Owing to the nature of the intervention, masking (blinding) was not possible.

### Statistical analysis

Data analyses were performed using SPSS 25.0 for Windows and interpreted on a 5% significance level. Differences in patient and disease characteristics between the MULTI_sh and TAU groups were analysed using independent *t*-tests (continuous variables) and χ² statistics (dichotomous variables). Participants whose change score for one measurement was missing were excluded from the analysis for that particular variable. Continuous variables were examined for normality, homogeneity and linearity as assumptions for *t*-tests and linear regression analyses by visual inspection of distributions, Q–Q and scatterplots and by comparing means with medians and standard deviations. If assumptions were violated, non-parametric tests were executed.

Multiple regression analyses were used to evaluate changes in outcomes between the MULTI_sh and TAU groups. Change scores on the outcome variables (*T*_2_–*T*_1_) were regressed on the treatment variable (model 1) and adjusted for the baseline value and different municipalities to account for potential regression to the mean and clustering within other sheltered housing locations. All analyses were primarily based on intention to treat. Additionally, per-protocol analyses were conducted.

To evaluate the MIDI determinants, we used the means and standard deviations, as well as the score per subscale, after recoding negatively stated items. Items to which ≥20% of the healthcare professionals and patients responded negatively (corresponding to ‘totally disagree/disagree’, score <3) were considered barriers and those to which ≥80% of healthcare professionals and patients responded positively (corresponding to ‘agree/totally agree’, score >3) were considered facilitators in the implementation of MULTI_sh. Proportions of score 3 were reported as neutral. A detailed description of the statistical analyses has been given elsewhere.^23^

## Results

**Fig. 1:**
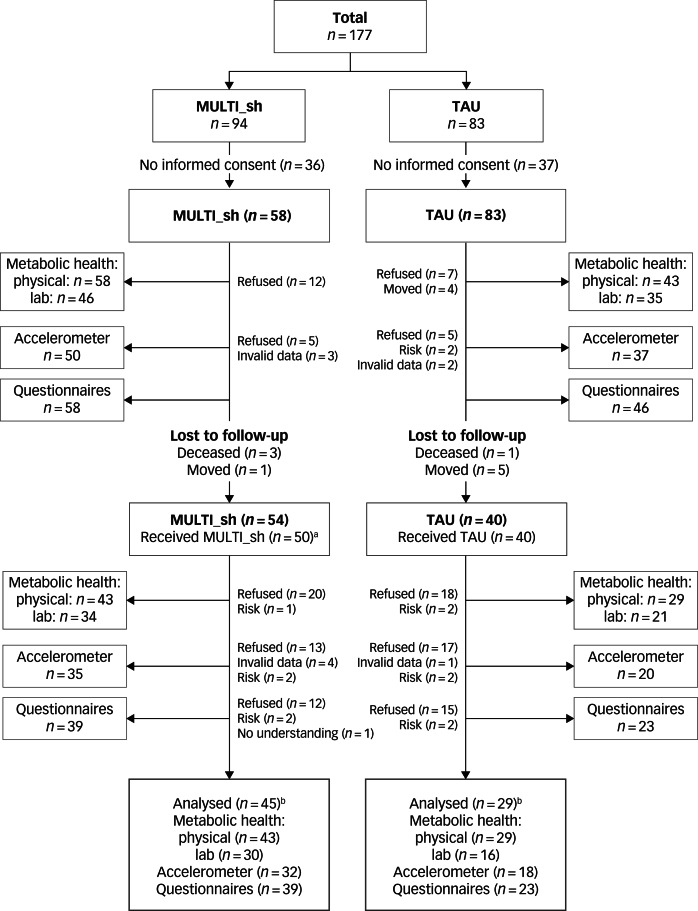
Flowchart including the number of participants per outcome and reasons for incomplete data on baseline and follow-up. MULTI_sh, multidisciplinary lifestyle-enhancing treatment for people with severe mental illness living in sheltered housing; TAU, treatment as usual; lab, laboratory tests. ^a^Four patients did not receive support by the sheltered housing teams for most of the study time because they were not around (e.g. partly lived elsewhere). ^b^Number of participants for whom both baseline and follow-up measurements of at least one outcome were available.

As shown in Fig. 1, out of 177 eligible patients, 74 (42%) could eventually be included in analyses (i.e. both baseline and follow-up measurements of at least one outcome available). Of the 104 patients who gave informed consent to participate in the study, 30 dropped out for analyses. They did not significantly differ in age, gender, diagnosis or baseline illness severity compared with patients included in the analyses. Of the patients included in the analyses, there were also no significant differences in patient and illness characteristics between participants in the MULTI_sh (*n* = 45) and the TAU groups (*n* = 29). The support of the sheltered housing healthcare professionals was limited for four patients because those patients were not around for most of the study time. Consequently, the per-protocol analyses were conducted for 41 and 29 participants in intervention and control conditions respectively. No adverse events were reported.

Of the 41 patients still involved in MULTI_sh at follow-up, 39 completed the semi-structured interview evaluating its implementation. Two dropped out because they refused or because the majority of the interview could not be completed. Of the 36 available healthcare professionals, 4 did not respond and 6 ended the questionnaire after a few items, resulting in responses from 26 participants for analyses.

### Health outcomes

There was little change in abdominal girth and other metabolic health outcomes in either the MULTI_sh or TAU groups (Table 1). Similarly, little change was observed in both groups for sedentary behaviour and physical activity, psychopathology and QoL. Accordingly, multiple regression showed no clinically and statistically significant between-group effects for any health outcomes. The largest effects in adjusted regression, found for psychological QoL (B = 1.42, β = 0.28, 95% CI −0.14 to 2.98), total activity (B = 0.39, β = 0.27, 95% CI −0.17 to 0.94) and sedentary behaviour (B = −3.42, β = 0.27, 95% CI −7.31 to 0.48), remained relatively small (supplementary Table 1, available at https://dx.doi.org/10.1192/bjo.2022.600). Per-protocol analyses resulted in different, statistically significant outcomes only for psychological QoL in favour of MULTI_sh (B = 1.59, β = 0.36, 95% CI 0.22–2.97, *P* = 0.02).

**Table 1:** Baseline characteristics and baseline and follow-up measurements of participants on metabolic health, sedentary behaviour and physical activity, psychopathology and quality of life (*n* = 74)

Characteristics/outcome/scale	MULTI_sh (*n* = 45)		TAU (*n* = 29)	
	Baseline	Follow-up	Baseline	Follow-up
Gender, *n* (%) male	29 (64.4)		14 (48.3)	
Age, mean (s.d.) years	51.5 (11.2)		45.7 (16.2)	
Diagnosis, *n* (%)^a^				
Schizophrenia and other psychotic disorders	24 (54.5)		17 (58.6)	
Pervasive disorders	8 (18.2)		3 (10.3)	
Mood disorders	6 (13.6)		5 (17.2)	
Anxiety disorders	3 (6.8)		3 (10.3)	
Personality disorders	2 (4.5)		1 (3.4)	
Delirium, dementia, and amnestic and other cognitive disorders	1 (2.3)		−	
Metabolic health, mean (s.d.)				
Abdominal girth, cm^b^	105.8 (17.4)	107.5 (14.9)	104.9 (17.1)	107.0 (16.1)
Weight, kg^c^	87.2 (20.7)	88.4 (19.0)	91.3 (20.5)	91.7 (19.5)
Systolic blood pressure, mmHg	126.7 (16.7)	136.9 (17.2)	125.1 (15.2)	136.7 (19.6)
Diastolic blood pressure, mmHg	80.0 (11.1)	84.8 (11.1)	79.1 (10.2)	80.9 (11.3)
Triglycerides, mmol/l^d^	2.1 (1.9)	2.0 (1.0)	2.0 (1.7)	1.8 (1.1)
HDL cholesterol, mmol/l^d^	1.2 (0.3)	1.1 (0.2)	1.3 (0.3)	1.3 (0.3)
Fasting glucose, mmol/l^e^	6.2 (3.0)	6.1 (2.2)	5.8 (1.4)	6.1 (2.2)
HbA_1c_, mmol/l^f^	39.2 (10.2)	39.8 (7.7)	38.8 (7.0)	38.7 (8.6)
Sedentary behaviour and physical activity, mean (s.d.)^f^				
TAC/h	30247.5 (16164.8)	30801.2 (18827.1)	29358.7 (17460.4)	26929.9 (11576.4)
Sedentary behaviour, % of weartime	79.9 (8.6)	79.9 (7.3)	82.3 (7.3)	82.7 (5.3)
Light physical activity, % of weartime	12.6 (5.7)	12.6 (5.0)	10.3 (3.5)	10.4 (3.4)
Moderate-to-vigorous physical activity, % of weartime	7.5 (4.9)	7.5 (5.4)	7.3 (4.6)	6.9 (3.7)
Wear time during measurement, h^g^	51.9 (11.6)	48.6 (11.8)	51.3 (10.4)	49.1 (10.4)
Psychopathology: BPRS-E, mean (s.d.)				
Total	42.3 (11.7)	40.3 (9.1)	47.0 (13.8)	41.8 (13.9)
Manic excitement/disorganisation	15.1 (5.2)	12.8 (3.9)	15.9 (7.5)	13.3 (5.5)
Depression/anxiety	12.6 (5.3)	13.0 (5.6)	15.7 (6.6)	13.2 (6.2)
Positive symptoms	8.0 (4.4)	8.0 (4.4)	8.2 (3.6)	8.2 (3.6)
Negative symptoms	11.0 (3.4)	10.7 (3.8)	12.2 (4.3)	12.1 (5.7)
Quality of life, mean (s.d.)				
EQ-5D index score (range 0–1)	0.7 (0.3)	0.8 (0.2)	0.6 (0.3)	0.7 (0.3)
WHOQoL-Bref domain scores (range 4–20)				
Physical	14.1 (2.7)	14.2 (2.3)	13.2 (2.9)	14.5 (2.0)
Psychological	13.6 (2.8)	14.5 (2.5)	12.8 (2.4)	13.0 (2.3)
Social	13.6 (3.1)	14.5 (3.3)	13.1 (3.3)	14.0 (2.2)
Environmental	14.9 (2.0)	15.7 (2.0)	14.4 (2.0)	14.9 (1.2)

MULTI_sh, multidisciplinary lifestyle-enhancing treatment for patients with severe mental illness living in sheltered housing; TAU, treatment as usual; BPRS-E, Brief Psychiatric Rating Scale Expanded Version; TAC/h, total activity counts per hour; EQ-5D, EuroQol five-dimension questionnaire; WHOQOL-Bref, World Health Organization Quality of Life Brief Version.

a. *n* = 44 for MULTI_sh and *n* = 29 for TAU, owing to missing diagnostic data.

b. *n* = 45 for MULTI_sh and *n* = 27 for TAU, owing to missing abdominal girth data.

c. *n* = 45 for MULTI_sh and *n* = 29 for TAU, owing to missing weight data.

d. *n* = 38 for MULTI_sh and *n* = 24 for TAU, owing to missing triglycerides and HDL cholesterol data.

e. *n* = 37 for MULTI_sh and *n* = 26 for TAU, owing to missing fasting glucose data.

f. *n* = 33 for MULTI_sh and *n* = 24 for TAU, owing to missing HbA_1c_ data.

g. *n* = 39 for MULTI_sh and *n* = 24 for TAU, owing to insufficient wear time of the accelerometer.

### Implementation

Table 2 shows the participant characteristics regarding the analysed implementation determinants. The question serving as a proxy for fidelity (descriptive norm) was answered by 26 healthcare professionals, of whom 35% (*n* = 9) indicated that half or less than half of their colleagues carried out MULTI_sh as intended. Of the patients who completed the MIDI interview (*n* = 39), ten answered that they could not correctly assess the question because their daily activity programme had not changed and/or they were not familiar with the programme as intended. Of the patients who did answer (*n* = 29), the majority (*n* = 15, 52%) estimated that less than half of the team carried out the programme as intended.

**Table 2:** Participant characteristics and scores for implementation determinants concerning MULTI_sh, healthcare professionals/patients and the organisation, with percentages for negative, neutral and positive responses^a^

	Healthcare professionals (*n* = 26)			Patients (*n* = 39)		
Gender, *n* (%) male	4 (15.4)			27 (69.2)		
Age, years, mean (s.d.)	41.6 (14.8)			50.1 (12.3)		
Diagnosis, *n* (%)						
Schizophrenia and other psychotic disorders				20 (51.3)		
Pervasive disorders				8 (20.5)		
Mood disorders				5 (12.8)		
Personality disorders				2 (5.1)		
Other disorders^b^				4 (10.3)		
Disciplines, *n* (%)						
Residential mentor (nurse, social worker)	21 (80.7)					
Residential mentor trainee	2 (7.7)					
Team leader	2 (7.7)					
Psychiatrist	1 (3.4)					
	% neg.	% neu.	% pos.	% neg.	% neu.	% pos.
Determinants of MULTI_sh	3.8	−	**96**.**2**	**20**.**5**	10.3	69.2
Procedural clarity	7.7	19.2	73.1			
Correctness	−	−	**100**.**0**			
Completeness	15.4	34.6	50.0	10.3	12.8	76.9
Complexity^c^	3.8	11.5	**84**.**6**	**31**.**4**	8.6	60.0
Congruence current method^d^	3.8	34.6	61.5	**20**.**6**	20.6	58.8
Observability^c^	15.4	65.4	19.2	**20**.**0**	20.0	60.0
Relevance for patient^c^	−	26.9	73.1	14.3	17.1	68.6
Determinants of healthcare professionals/residents	3.8	−	**96**.**2**	18.4	−	**81**.**6**
Personal benefit^e^	**30**.**8**	34.6	34.6	19.4	13.9	66.7
Personal disadvantage^f^	19.2	23.1	57.7	6.1	15.2	78.8
Outcome expectations^d^	3.8	−	**96**.**2**	17.6	14.7	67.6
Task perception^e^	−	−	**100**.**0**	**22**.**2**	19.4	58.3
Patient satisfaction^c^	3.8	30.8	65.4	5.7	14.3	**80**.**0**
Patient/healthcare professional collaboration^d^	**26**.**9**	38.5	34.6	**29**.**4**	20.6	50.0
Social support^c^	11.5	3.8	**84**.**6**	11.4	8.6	**80**.**0**
Descriptive norm (1–7)^g^	19.2	15.4	65.4	**51**.**7**	10.3	37.9
Subjective norm^e^	3.8	−	**96**.**2**	**47**.**2**	5.6	47.2
Self-efficacy	7.7	7.7	**84**.**6**			
Knowledge	3.8	11.5	**84**.**6**			
Awareness of the content of the treatment (1–4)^h^	3.8	96.2	−	**48**.**7**	51.3	−
Determinants of the organisation	15.4	7.7	76.9			
Formal ratification by management (no/yes)	**26**.**9**	−	73.1			
Replacement when healthcare professionals leave	19.2	46.2	34.6			
Healthcare professionals’ capacity	**26**.**9**	30.8	42.3			
Financial resources	**23**.**1**	42.3	34.6			
Time available	15.4	46.2	38.5			
Materials, resources and facilities	7.7	26.9	65.4			
Coordinator (no/yes)	11.5	−	**88**.**5**			
Organisational changes	**30**.**8**	23.1	46.2			
Information accessible about the use of innovation	15.4	34.6	50.0			
Performance feedback	7.7	26.9	65.4			

MULTI_sh, multidisciplinary lifestyle-enhancing treatment for patients with severe mental illness living in sheltered housing; neg., negative response (score <3); neu., neutral response (score 3); pos., positive response (score >3). Reported barriers (≥20% negative response) and facilitators (≥ 80% positive response) are shown in bold.

a. Scores could range from 1 to 5 unless noted otherwise in parentheses, and higher mean scores reflect a more positive contribution to the implementation of MULTI_sh.

b. Anxiety disorder (*n* = 1), delirium, dementia, and amnestic and other cognitive disorders (*n* = 1), alcohol-related disorders (*n* = 1), somatoform disorders (*n* = 1).

c. *n* = 35 for patients, owing to missing data.

d. *n* = 34 for patients, owing to missing data.

e. *n* = 36 for patients, owing to missing data.

f. *n* = 33 for patients, owing to missing data.

g. *n* = 29 for patients, owing to missing data; calculated to negative (scores of 1–3), neutral (4) and positive (5–7).

h. Calculated to negative (scores of 1–2) and positive (3–4).

#### Barriers

As can be seen in Table 2, healthcare professionals did not experience any barriers regarding MULTI_sh itself. Patients identified programme complexity, congruence with the current method (‘the lifestyle treatment fits well with how we aim to work on the ward’) and observability of results as barriers. Of the implementation determinants related to the healthcare professionals and patients themselves, a lack of personal benefits, particularly the negative experience of the time it takes to engage patients and challenges in patient cooperation, was identified as a barrier for healthcare professionals. Task perception (‘I think it is part of my treatment to improve my lifestyle’), the cooperation of the healthcare professionals (how well the nurses implemented MULTI_sh) and awareness of the content of the treatment (almost half of the patients did not recognise the method at all or recognised just a few components) were identified as barriers for patients. Organisational implementation determinants considered barriers by healthcare professionals were formal ratification by management (i.e. limited formal agreements have been made by management about the use of MULTI_sh within the organisation), healthcare professionals’ capacity, financial resources and organisational changes (which were mainly related to insufficient healthcare professionals’ capacity).

#### Facilitators

All the healthcare professionals believed that MULTI_sh was based on factually correct knowledge, and the majority did not think it was complicated. For patients, there were no facilitators related to MULTI_sh itself. Overall, for implementation determinants related to the user, healthcare professionals responded positively. The vast majority of the determinants were considered to be facilitators, such as outcome expectations, task perception (‘I find working in accordance with MULTI_sh my responsibility’), social support (‘I can count on adequate assistance’), self-efficacy and knowledge (‘I have enough knowledge to be able to use MULTI_sh’). The patients scored MULTI_sh positively on satisfaction and social support, where most help was expected from healthcare professionals (85.7%) and family members (71.4%). The only facilitator identified at the organisational level was having one person coordinate the implementation at each site.

## Discussion

The current study aimed to evaluate the effectiveness of MULTI_sh in improving both physical and mental health, as well as whether MULTI_sh was implemented as intended, and to identify associated implementation barriers and facilitators. Owing to the high drop-out in measurements, the study is too underpowered to make any statements on the statistical significance of between-group effects. Nevertheless, no clinically significant changes were seen in either group, and inadequate implementation was observed.

Although evidence on the efficacy of lifestyle interventions for physical health outcomes in SMI is inconsistent,^9,43^ finding no change in any outcome goes against an emerging body of evidence.^10–12,24^ According to the results on implementation factors, the most likely explanation seems to be inadequate implementation of MULTI_sh, which makes it impossible to make any statements about the effects of MULTI_sh as intended. One-third of the healthcare professionals indicated that, at most, half of them conducted MULTI_sh as intended. Most patients estimated that less than half of the team carried out the programme as intended, and ten patients reported that their daily activity programme did not change and/or they were unfamiliar with the programme description. Also, if MULTI_sh had been implemented to its full extent, one would have expected to observe increases in physical activity and decreases in sedentary behaviour within the MULTI_sh group (e.g.^20^), as increasing physical activity is one of the critical elements of MULTI_sh. However, no clinically significant improvements in physical activity and sedentary behaviour were observed.

Healthcare professionals indicated that the main implementation barriers were (in)directly linked to organisational factors involving a shortage of healthcare professionals, lack of available time, difficulty getting the patients involved and unfamiliarity with formal agreements made by their management regarding the utility of MULTI_sh. The organisational barriers also correspond with the start of MULTI_sh, as the period between baseline measurement and implementation was longer than intended owing to turnover of healthcare professionals and turbulence within the organisation. This may already have contributed to implementation failure. Previous studies reported that the above-mentioned problems can have a negative impact on the support of lifestyle-related behaviour by mental health nurses and that support from management is essential.^44–46^ The main barriers experienced by patients match those of healthcare professionals, with their reported complexity of participating in MULTI_sh and a lack of others expecting them to do so. In line with these factors, patients’ illness severity and need for support to overcome difficulties related to their illness were identified as barriers.

Additionally, it appears that difficulties in multidisciplinary cooperation affected the implementation. In contrast to the clinical setting (e.g.^20^), where activity coordinators, mental health professionals and dietitians were present on site, in the sheltered housing setting these were not part of the regular healthcare professionals’ team. Previously, MULTI in the in-patient setting improved health-related outcomes, although there were many organisational barriers.^20–23^ However, the descriptive norm (i.e. how well colleagues implemented MULTI) was perceived to be considerably more positive by both healthcare professionals (81% positive, versus 65% in the current study) and patients (79% *v*. 38%).^23^ More intensive team cooperation due to the 24 h in-patient care with more staff, including qualified professionals with expertise on lifestyle factors, might have helped them improve despite organisational barriers. However, that study also found that these organisational factors needed to be improved to continue MULTI and to sustain the health benefits.^23^ In the current study, after 12 months of receiving MULTI_sh, there was still little collaboration and contact between some sheltered housing facilities and appropriate social services. Apart from showing how challenging setting up such collaborations in a community setting can be, this also stresses that the teams require support from qualified professionals with expertise in lifestyle factors, as this has been identified several times as being critical to success.^12,47,48^ Therefore, one could argue that the teams should have received more support during implementation to overcome their struggles. However, our study aimed to test the current way of implementation in real-world conditions.

### Implications

A crucial facilitating factor identified was that most patients and all healthcare professionals indicated that they believed improving patients’ lifestyle should be part of treatment.

Our findings indicate that intensive implementation strategies, especially at the organisational level, are warranted to successfully implement a multidisciplinary multi-component lifestyle intervention in sheltered housing. An implementation strategy is a method or technique that can stimulate the implementation of an intervention by targeting barriers.^18^ Besides prioritising the topic and addressing healthcare professionals’ capacity and lack of time, integrating professionals qualified to support changing lifestyle behaviour in mental healthcare seems key to helping patients and the existing workforce. Multiple parties are stakeholders in this, such as the management of the mental healthcare organisation itself, their healthcare professionals and patients, the allied health professionals who can support patients and healthcare professionals in making lifestyle changes (in The Netherlands, this support falls within the financial domain of the municipalities) and the health insurance companies (regarding the reimbursement of the professionals needed). For example, a collaborative strategy such as setting up a consortium or coalition with a representation of stakeholders intends to align the perspectives and context of parties and promote development of mutual competence.^18^ This would suit the goal of achieving a more integrative approach, which could help healthcare professionals to take more time with more organisational support to implement MULTI_sh. In turn, this could also help patients to overcome barriers and convert their predominantly positive attitude, recognition and willingness into actual change. Although the above-mentioned barriers seemed key to the failed implementation and are therefore a priority in finding suitable implementation strategies, developing strategies to address other barriers simultaneously (e.g. improving the information about MULTI_sh for patients) can contribute to better implementation. However, to unravel in more detail what strategies are required to improve the implementation of MULTI_sh needs more work. Overall, for organisations who want to improve the lifestyle and poor health of people with SMI, it is essential to be aware that success depends heavily on the priority of the topic within the organisation and the support for staff and patients to enable them to implement it.

### Limitations

First, the study was considerably underpowered (i.e. *n* = 45 in the MULTI_sh group and *n* = 29 in the TAU, instead of *n* = 84 each) owing to a high drop-out rate on measurements. This reflects the ongoing challenge of involving patients with SMI in evaluations in real-world settings. To achieve and maintain a larger sample size in such studies, it is vital to keep communicating their relevance and benefit for improving healthcare and to search for methods of measuring that pose the lowest burden on participants to minimise participation refusal. Second, owing to the naturalistic setting of the study and the nature of the intervention, patients were not randomised on an individual level. Also, there might be bias among healthcare professionals regarding the implementation questionnaire, as the least motivated may not have responded, despite the encouragement to participate regardless of attitude towards the topic and intervention, as we aimed to capture the whole spectrum. Third, in the context of comparative tests, it is also worthwhile mentioning that because multiple outcomes were tested, statistical significance may be misleading. However, instead of a binary report of significance versus non-significance, *P*-values are presented so that the readers can determine the statistical significance according to their standard. In addition, we considered that the magnitude of the effect is more important than statistical significance and that the magnitude of the effect is not affected by multiple testing. Moreover, it would be of value in future studies to include insight into changes in outcomes of personal importance to participants, which may differ from the significance of outcomes measured across the group. This can reveal other favourable outcomes and is essential in the context of engagement. A last possible limitation is that not all the facets that MULTI_sh focuses on can be assessed from our results. For example, we did not study whether the patients’ dietary pattern improved.

### Strengths

A notable strength of the study was the controlled design in a naturalistic environment, which was essential to external validity. Other research studying the effects of lifestyle interventions has often been performed under ideal controlled conditions, which are crucial to study efficacy but unlikely to reflect the average level of available time and resources for interventions in daily practice (e.g.^17^). The current study represents the routine mental healthcare in sheltered housing facilities, including challenges such as everyday problems, limited available time and turnover of healthcare professionals. In this context, the effectiveness-implementation hybrid design is of added value to gain insight into implementation-related factors and strongly contributes to preventing type 3 error (i.e. rejecting the hypothesis for the wrong reasons), especially in this case of negative findings.

Furthermore, the study sample was representative of daily clinical practice, as patients were included regardless of gender, age or illness severity. The present study contributes to filling the gap between research and practice, by demonstrating the use of methods to improve the external validity to create appropriate evidence which supports decision-making for clinicians and healthcare professionals at management level in real-world settings.^16,17,49,50^

### Further research

This study revealed that MULTI_sh was not implemented as intended and that more intensive implementation strategies at an organisational level are warranted. Further studies considering implementation factors in applying lifestyle interventions in sheltered housing are encouraged as the challenge of improving the mental and physical health of people with SMI remains.

## Data Availability

The data that support the findings of this study are available from the corresponding author on reasonable request.

## Supplementary material

For supplementary material accompanying this paper visit http://doi.org/10.1192/bjo.2022.600. click here to view supplementary material
