# Supplementary material for: Evaluating the implementation of a multidisciplinary lifestyle intervention for people with severe mental illness in sheltered housing: effectiveness-implementation hybrid randomised controlled trial
Source: BJPsych Open. 2022 Nov 22;8(6):e201. doi: 10.1192/bjo.2022.600 (PMC9707511; doi:10.1192/bjo.2022.600)
Supplement: Supplementary file 1 [file S2056472422006007sup001.pdf]

Table S1. Multiple regression estimating treatment effect between MULTI_sh compared to TAU on physical and mental health outcomes (n=74).

	Model 1				Model 2			
Outcome (scale)	B	β	(95% CI)	p	B	β	(95% CI)	p
Metabolic health								
Abdominal girth (cm)[a]	-2.22	-0.09	(-8.66-4.21)	0.49	-2.93	-0.12	(-10.73-4.86)	0.45
Weight (kg)[b]	0.60	0.03	(-4.82-6.03)	0.82	-1.87	-0.08	(-8.83-5.09)	0.59
Systolic blood pressure (mmHg)	-1.39	-0.04	(-10.29-7.51)	0.76	-8.84	-0.24	(-19.01-1.32)	0.09
Diastolic blood pressure (mmHg)	3.65	0.17	(-1.50-8.79)	0.16	0.003	<0.01	(-6.05-6.05)	0.99
Triglycerides (mmol/l)[c]	0.17	0.05	(-0.79-1.13)	0.72	0.01	<0.01	(-0.70-0.72)	0.98
HDL cholesterol (mmol/l)[c]	0.02	0.05	(-0.091-0.13)	0.72	0.08	0.21	(-0.03-0.18)	0.15
Fasting glucose (mmol/l)[d]	-0.34	-0.10	(-1.32-0.63)	0.48	0.27	0.08	(-0.84-1.37)	0.63
HbA1c (mmol/l)[e]	-1.19	-0.15	(-3.65-1.28)	0.34	1.47	0.19	(-1.69-4.62)	0.35
Sedentary behavior and physical activity[f]								
TAC/h	0.24	0.15	(-0.21-0.68)	0.29	0.39	0.27	(-0.17-0.94)	0.17
Sedentary behavior (%)	-0.92	-0.07	(-4.60-2.77)	0.62	-3.42	-0.27	(-7.31-0.48)	0.08
Light physical activity (%)	0.35	0.04	(-2.26-2.95)	0.79	1.99	0.22	(-0.92-4.91)	0.18
Moderate-to-vigorous physical activity (%)	0.57	0.08	(-1.58-2.72)	0.60	1.20	0.16	(-1.30-3.70)	0.34
Psychopathology: BPRS-E total[g]	2.24	0.09	(-4.45-8.93)	0.51	-3.89	-0.15	(-11.33-3.55)	0.30
Manic excitement/ disorganization	-0.73	-0.06	(-3.76-2.32)	0.63	-1.74	-0.15	(-4.80-1.32)	0.26
Depression/ anxiety	3.05	0.25	(0.03-6.06)	0.05	0.49	0.04	(-3.36-4.34)	0.80
Positive symptoms	0.08	0.01	(-2.07-2.23)	0.94	-0.83	-0.10	(-2.78-1.13)	0.40
Negative symptoms	-0.15	-0.02	(-2.52-2.23)	0.90	-1.13	-0.12	(-4.18-1.92)	0.46
EQ-5D[g]	-0.08	-0.12	(-0.24-0.09)	0.36	0.09	0.14	(-0.07-0.24)	0.28
WHOQoL-Bref domains[g]								
Physical	-1.19	-0.23	(-2.51-0.12)	0.07	-0.14	-0.03	(-1.48-1.20)	0.84
Psychological	0.62	0.12	(-0.67-1.90)	0.34	1.42	0.28	(-0.14-2.98)	0.07
Social	-0.20	-0.03	(-2.01-1.62)	0.83	0.24	0.03	(-1.80-2.27)	0.81
Environmental	0.52	0.12	(-0.55-1.59)	0.33	0.96	0.23	(-0.20-2.13)	0.10

Model 1: crude. Model 2: adjusted for baseline measurement and municipalities.

MULTI_sh: Multidisciplinary Lifestyle-enhancing Treatment for patients with severe mental illness living in sheltered housing, TAU: Treatment as Usual, BPRS-E: Brief Psychiatric Rating Scale, PA: Physical Activity, TAC/h: Total Activity Counts per hour (standardized), QoL, EQ-5D: EuroQol five-dimension questionnaire, WHOQoL-Bref: World Health Organization Quality of Life questionnaire brief version

[a] N = 63, due to missing abdominal girth data.
[b] N = 70, due to missing weight, systolic and diastolic data.
[c] N = 46, due to missing triglycerides and HDL cholesterol data
[d] N = 49, due to missing fasting glucose data.
[e] N = 42, due to missing HbA1c data
[f] N = 50, due to insufficient wear time of the accelerometer.
[g] N = 62, due to missing BPRS, EQ-5D and WHOQoL-Bref data
